# Circulating Cell-Free DNA and Circulating Tumor Cells as Prognostic and Predictive Biomarkers in Advanced Non-Small Cell Lung Cancer Patients Treated with First-Line Chemotherapy

**DOI:** 10.3390/ijms18051035

**Published:** 2017-05-11

**Authors:** Simona Coco, Angela Alama, Irene Vanni, Vincenzo Fontana, Carlo Genova, Maria Giovanna Dal Bello, Anna Truini, Erika Rijavec, Federica Biello, Claudio Sini, Giovanni Burrafato, Claudia Maggioni, Giulia Barletta, Francesco Grossi

**Affiliations:** 1 Lung Cancer Unit, IRCCS AOU San Martino—IST Istituto Nazionale per la Ricerca sul Cancro, L.go R. Benzi 10, 16132 Genova, Italy; simona.coco@hsanmartino.it (S.C.); angela.alama@hsanmartino.it (A.A.); carlo.genova1985@gmail.com (C.G.); mariagiovanna.dalbello@hsanmartino.it (M.G.D.B.); anna.truini@yale.edu (A.T.); erika.rijavec@hsanmartino.it (E.R.); febiello@gmail.com (F.B.); audiosini@tiscali.it (C.S.); g_burrafato@libero.it (G.B.); claudia_87m@yahoo.it (C.M.); giulia.barletta@yahoo.it (G.B.); francesco.grossi@hsanmartino.it (F.G.); 2 Clinical Epidemiology Unit, IRCCS AOU San Martino—IST Istituto Nazionale per la Ricerca sul Cancro, L.go R. Benzi 10, 16132 Genova, Italy; vincenzo.fontana@hsanmartino.it; 3 Department of Internal Medicine and Medical Specialties (DIMI), University of Genova, IRCCS AOU San Martino—IST Istituto Nazionale per la Ricerca sul Cancro, L.go R. Benzi 10, 16132 Genova, Italy

**Keywords:** liquid biopsy, circulating free DNA, circulating tumor cells, non-small cell lung cancer (NSCLC), biomarkers, chemotherapy

## Abstract

Cell-free DNA (cfDNA) and circulating tumor cells (CTCs) are promising prognostic and predictive biomarkers in non-small cell lung cancer (NSCLC). In this study, we examined the prognostic role of cfDNA and CTCs, in separate and joint analyses, in NSCLC patients receiving first line chemotherapy. Seventy-three patients with advanced NSCLC were enrolled in this study. CfDNA and CTC were analyzed at baseline and after two cycles of chemotherapy. Plasma cfDNA quantification was performed by quantitative PCR (qPCR) whereas CTCs were isolated by the ScreenCell Cyto (ScreenCell, Paris, France) device and enumerated according to malignant features. Patients with baseline cfDNA higher than the median value (96.3 *hTERT* copy number) had a significantly worse overall survival (OS) and double the risk of death (hazard ratio (HR): 2.14; 95% confidence limits (CL) = 1.24–3.68; *p*-value = 0.006). Conversely, an inverse relationship between CTC median baseline number (6 CTC/3 mL of blood) and OS was observed. In addition, we found that in patients reporting stable disease (SD), the baseline cfDNA and CTCs were able to discriminate patients at high risk of poor survival. cfDNA demonstrated a more reliable biomarker than CTCs in the overall population. In the subgroup of SD patients, both biomarkers identified patients at high risk of poor prognosis who might deserve additional/alternative therapeutic interventions.

## 1. Introduction

Non-small cell lung cancer (NSCLC) accounts for about 75–80% of all lung cancers [1,2] and despite improved diagnostic techniques, the great majority of NSCLC patients (70%) present advanced stage tumors at diagnosis and there is a 5-year survival rate of less than 5% [3,4]. The mainstay of care for patients affected by advanced NSCLC in absence of actionable driver mutations is first-line platinum-based chemotherapy; however, the prognosis of treated patients remains dismal, with a median survival of about one year [5,6]. Presently, no specific biomarker that helps clinicians determine prognosis and monitor patients’ response during treatment with chemotherapy has been identified in NSCLC, but much interest has been focused on biomarkers identified by liquid biopsy.

Liquid biopsy is a non-invasive blood test that detects tumor-derived nucleic acids (cell-free tumor DNA (cfDNA) and microRNAs) as well as circulating tumor cells (CTCs) shed by the tumor into the bloodstream [7,8]. Specifically, cfDNA is released into the circulation from primary or metastatic cancers through cell death mechanisms including apoptosis, necrosis, phagocytosis and lysis of tumor cells, thus representing an indicator of cellular turnover [9]. Conversely, CTCs are spread from the tumor into the peripheral blood, playing an important role in the development of metastasis [10]. Since blood samples can be obtained at different times during treatment, the clinical response as well as the emergence of drug resistance can be monitored in real time.

Recently, the Food and Drug Administration (FDA) approved the CellSearch system (Janssen Diagnostics, Raritan, NJ, USA) for CTC detection and enumeration of the major malignancies such as breast, colon and prostate cancer [11,12,13]. This methodology involves antibodies directed against specific epithelial tumor markers; however, filtration by size systems might be more suitable to detect CTCs, irrespective of cell surface-markers [14,15,16].

The potential value of liquid biopsy in lung cancer has been recently underlined as being tissue accessibility, which is often challenging. To date, a number of studies have highlighted the relevance of cfDNA and CTCs as NSCLC biomarkers [17,18], however, to the best of our knowledge, their simultaneous assessment has not yet been uncovered. In this study, we sought to evaluate the role of cfDNA quantification and CTC enumeration, separately or conjunctionally, in predicting response to treatment and survival in a cohort of advanced NSCLC patients receiving first line platinum-based chemotherapy.

## 2. Results

### 2.1. Study Population

Seventy-three patients affected by advanced stage IIIB–IV NSCLC who were candidates for palliative first-line platinum-based chemotherapy were considered eligible and enrolled in the study. The characteristics of the patients considered in the analyses are summarized in Table 1. The median age at first cycle (start of chemotherapy) was 67 years, the majority of patients were males (68.5%), the frequency of adenocarcinoma histology was 80.8% and Eastern Cooperative Oncology Group Performance Status (ECOG PS) of 1–2 accounted for 71.2% of cases. In addition, 92% of patients were current/former smokers. Among the enrolled patients, none harbored sensitizing mutations of epidermal growth factor receptor (*EGFR*); more specifically, three patients had mutations in exon 20 (resistant to EGFR tyrosine kinase inhibitors, and were therefore considered candidates for first-line chemotherapy [19]. In addition, no patients harbored anaplastic lymphoma kinase (*ALK*) rearrangements. Notably, all the patients but one had stage IV (metastatic) disease; the single patient with stage IIIB disease was included in the study due to the extension of the disease (T4N2), which excluded loco-regional treatments or combined modalities (such as chemo-radiation), leaving platinum-based chemotherapy as the only available therapeutic option. The patients affected by squamous cell carcinoma received a median of four cycles of chemotherapy with gemcitabine and a platinum-derivate (eight patients received cisplatin and four received carboplatin), while the patients affected by adenocarcinoma received a median of four cycles of chemotherapy with pemetrexed and a platinum-derivate (23 patients received cisplatin and 37 received carboplatin; one patient who was initially a candidate for cisplatin was switched to carboplatin after the first cycle due to a creatinine increase). Among patients treated with pemetrexed, 32 received at least one cycle of maintenance (range: 1–22; median: four cycles). Imaging with computed tomography (CT-scan) as well as blood withdrawals for liquid biopsy (cfDNA and CTCs) were performed at baseline (before chemotherapy) and after two and four cycles of chemotherapy. For one patient, the evaluation of cfDNA at baseline was not feasible due to plasma hemolysis. Blood samples were no longer collected in patients with unacceptable toxicity and deterioration of clinical conditions. For the above-mentioned reasons, the first assessment of cfDNA and CTCs after two cycles of chemotherapy (first evaluation) was feasible in 47 patients (two plasma samples were hemolyzed and not suitable for analysis) and 49 patients, respectively, whereas after four cycles of chemotherapy (second evaluation) only 21 (five plasma samples were not suitable) and 26 patients were evaluable for cfDNA and CTC analyses, respectively. Due to the small number of patients completing four cycles of chemotherapy, only cfDNA and CTC values at first evaluation by Response Evaluation Criteria in Solid Tumors (RECIST) were taken into account for the analyses.

The median follow-up time of progression free survival (PFS) of 70 evaluable patients (not evaluable in three) was 4.7 months (range: 0.9–26.1) while the median follow-up time of overall survival (OS) of the whole population of 73 patients was 8.0 months (range: 1.0–49.9). The best overall response (BOR) according to RECIST criteria was considered for comparative analyses with cfDNA and CTCs, reporting 16 patients (21.9%) with partial response (PR), 39 patients (53.4%) with stable disease (SD), 16 patients (21.9%) with progressive disease (PD) and two that were not evaluable (2.8%).

### 2.2. Role of Clinical Parameters and Circulating Biomarkers in Prognosis

**Figure 1:**
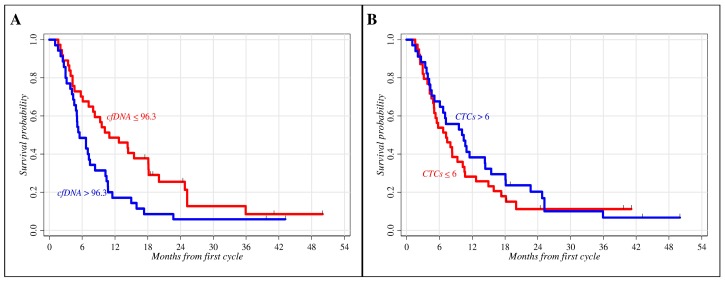
Overall survival according to circulating biomarkers at baseline by Kaplan–Meier analysis. Non-small cell lung cancer (NSCLC) patients were divided into two groups according to median baseline values. (**A**) Cell-free DNA (cfDNA): 96.3 human Telomerase Reverse Transcriptase (*hTERT*) copy number (*p*-value = 0.019); (**B**) Circulating tumor cells (CTCs): 6 CTCs/3 mL of blood (*p*-value = 0.402).

The prognostic role of clinical parameters (age, gender, histology, and ECOG PS) and circulating biomarkers (cfDNA and CTCs) in advanced NSCLC patients treated with first-line chemotherapy was evaluated by univariate Kaplan–Meier survival analyses. The median baseline plasma cfDNA value of 96.3 human Telomerase Reverse Transcriptase (*hTERT*) copy number (range: 16.7–1968.2) and the median baseline number of 6 CTCs/3 mL of blood (range: 0–50) were used as cut-off values to categorize patients into prognostic subgroups with potentially different outcomes (Figure 1). A minimal inverse correlation between cfDNA and CTCs at baseline was identified (Pearson correlation after log-transformation = −0.21, *p*-value = 0.08). Conversely, no significant associations were found among the cfDNA and CTCs values and some clinical features known to be linked to poor prognosis such as histology (adenocarcinoma vs. squamous cell carcinoma: cfDNA *p*-value = 0.706; CTCs *p*-value = 0.905) and the metastatic status (thoracic district vs. extra-pulmonary: cfDNA *p*-value = 0.991; CTCs *p*-value = 0.406; evidence vs. no evidence of brain metastases: CTCs *p*-value = 0.697). The only significant correlation was observed between the baseline cfDNA values and brain tumor metastases (*p*-value = 0.041).

As shown in Table 2, age at first cycle of chemotherapy was the only significant indicator of prognosis among the clinical factors reporting a survival probability at 18 months of 32.0% for patients younger than 67 compared to 14.0% for those older than 67 (*p*-value = 0.050). Regarding circulating biomarkers, patients with baseline cfDNA *hTERT* copy number ≤ 96.3 had a survival probability at 18 months of 38.0% vs. 9.0% for patients with higher cfDNA values (*p*-value = 0.019). No significant difference between patients with CTCs ≤ 6 and those with CTCs > 6 was observed, although patients with higher CTCs had a slightly longer median follow-up than patients with CTCs below the median value (10.3 vs. 7.2 months, respectively) and a survival probability at 18 months of 29.0% for patients with higher CTCs respect to 18.0% with lower CTCs (*p*-value = 0.402) showing an inverse correlation respect to cfDNA. In addition, no remarkable difference in PFS was found for either cfDNA or CTCs (data not shown).

Successively, the relationship between circulating biomarkers and prognosis, in terms of PFS and OS, was evaluated by the Cox multiple regression model (Table 3).

**Figure 2:**
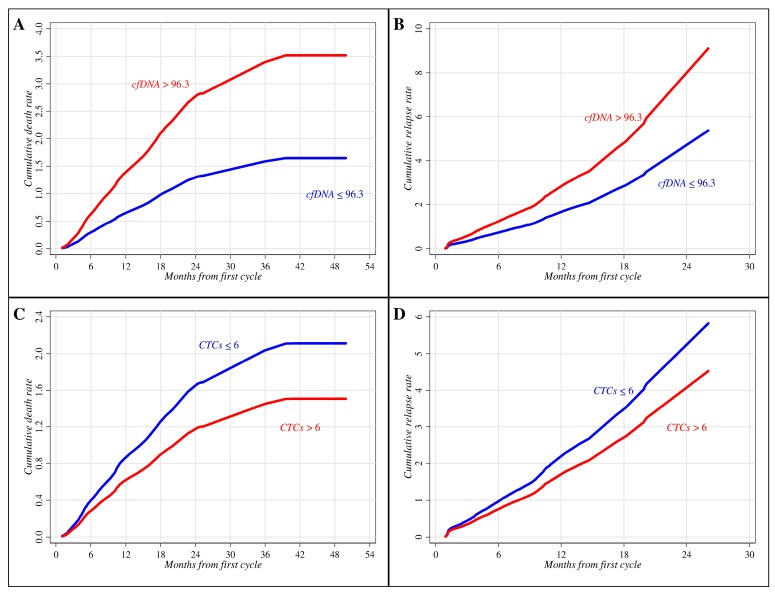
Curves of cumulative death and relapse rates according to cfDNA and CTCs by Cox regression modeling. Death (**A**) and relapse (**B**) rates estimated by cfDNA *hTERT* copy number. Death (**C**) and relapse (**D**) rates estimated by CTC number.

In this context, cfDNA and CTCs were first fitted to survival data separately (i.e., the two biomarkers are fitted separately to survival data through two distinct equations) and then jointly (i.e., both biomarkers are included in the same regression equation) in order to assess the degree of reciprocal influence. Separate Cox regression analysis confirmed the prognostic role of cfDNA (Figure 2). Indeed, the risk of death in patients with cfDNA > 96.3 was significantly higher than in patients with cfDNA ≤ 96.3 (hazard ratio (HR): 2.14; 95% confidence limits (CL) = 1.24–3.68; *p*-value = 0.006) (Figure 2A), and a similar result was obtained when relapse was used as an endpoint (HR: 1.70; 95% CL = 1.02–2.83; *p*-value = 0.040) (Figure 2B). Conversely, no significant association with OS and PFS was found by CTC enumeration, although a worse cumulative death rate was observed in patients with CTCs ≤ 6 (Figure 2C,D). Results obtained through the joint regression analysis showed a moderate influence of the two biomarkers in predicting PFS and OS, providing equivalent death and relapse rates (higher for patients with cfDNA > 96.3 and lower for patients with CTCs ≤ 6) and homogeneous statistical results (Table 3). Moreover, these findings suggest a substantially independent reciprocal prognostic role of cfDNA and CTCs in these NSCLC patients treated with chemotherapy.

Finally, the Cox regression was applied to the 39 evaluable patients experiencing SD at BOR. In this subgroup, substantial changes were observed when separate and joint biomarker models were fitted to OS data (Table 4). In the separate analysis, cfDNA confirmed its prognostic effect (HR: 2.32; 95% CL = 1.01–2.53; *p*-value = 0.047). Similarly, although with borderline significance, a higher CTC number (>6) was able to identify patients with a better OS (HR: 0.47; 95% CL = 0.22–1.03; *p*-value = 0.058). These findings suggest that in the subset of SD patients, higher cfDNA content and lower CTC number may differentiate subjects at a higher risk of early death. In the joint modeling, both biomarkers lost their significant prognostic impact but a higher risk of poor prognosis in patients with cfDNA above vs. below the median value (HR: 1.87 vs. HR: 1.00, respectively) and CTCs below vs. above the median value (HR: 1.00 vs. HR: 0.59, respectively) was retained (Table 4).

### 2.3. Circulating Biomarkers and Treatment

The variations of cfDNA plasma content and CTC enumeration during chemotherapy were analyzed in relation to the patients’ BOR estimated by RECIST. There were 47 and 49 patients with cfDNA and CTC determinations, respectively, who were evaluable for the analysis. Taking into consideration baseline measurements, a decreasing non-significant trend in geometric mean (GM) values of cfDNA according to a worsening of clinical conditions was found after two cycles of chemotherapy. In fact, a cfDNA GM equal to 46.3 (95% CL = 16.6–129.3), 69.2 (95% CL = 26.7–179.5) and 82.4 (95% CL = 27.1–250.8) *hTERT* copy numbers were evidenced in patients experiencing PR, SD and PD, respectively. Similarly, lower CTC GM levels were found among patients with PR (2.78; 95% CL = 1.19–6.52) and SD (2.73; 95% CL = 1.16–6.45) when compared to patients with PD (4.08; 95% CL = 1.33–12.50).

These findings suggest that, although chemotherapy has reduced the whole cfDNA and CTC burdens, the higher values of circulating biomarkers in PD patients might indicate reduced responsiveness to treatment.

## 3. Discussion

Non-small cell lung cancer is still diagnosed at advanced stage, when the OS rate is very poor; therefore, the identification of novel prognostic indicators and predictive factors remains a top priority. The application of liquid biopsy, as a non-invasive test, represents a promising tool to identify prognostic/predictive biomarkers such as cfDNA, miRNAs and CTCs [7]. Despite circulating cell-free miRNAs as well as encapsulated exosome miRNAs are emerging biomarkers [8,20], but they are still far from becoming clinically useful markers [21]. Conversely, numerous efforts have been made in the isolation and quantification of cfDNA and CTCs in order to improve cancer diagnosis, prognosis, as well as predict treatment efficacy [22]. A number of studies have been carried out to establish the most reliable cfDNA and CTC determinations in advanced NSCLC patients treated with chemotherapy, although studies evaluating their simultaneous role are presently lacking [17,18].

We evaluated the significance of these biomarkers both in separate and joint analyses in a cohort of advanced NSCLC patients receiving first line chemotherapy. Despite that the study population might be seen as heterogeneous on the basis of clinical variables such as histology, performance status, or stage (IIIB–IV), it should be considered that all patients had non-oncogene-driven advanced NSCLC, they were treatment-naïve and all were candidates for the same therapeutic approach (platinum-based chemotherapy). More specifically, only one patient had stage IIIB disease, which was too extended for combinations including chemotherapy and radiation therapy; furthermore, as reported in Table 2, gender, histology, and ECOG PS were not associated with statistically significant differences in terms of OS.

Baseline cfDNA content below the median value (96.3 *hTERT* copy number) demonstrated a significant prognostic indicator of OS in our cohort of treated NSCLC patients in simple analysis. This effect was further confirmed by Cox multiple regression models for both OS and PFS. Our results are in line with previous studies examining the prognostic role of cfDNA in advanced NSCLC patients undergoing chemotherapy. In particular, in the meta-analyses by Ai et al. [17], six studies evaluated the impact of cfDNA concentration on the OS of advanced NSCLC patients (stage III–IV) treated with chemotherapy. All the studies [23,24,25,26,27,28], except one [29], demonstrated that higher levels of cfDNA were significantly associated with poorer OS. In addition, we found that among the fraction of patients reporting SD at BOR, the baseline cfDNA retained its prognostic role by discriminating patients at a higher risk of poor survival. It should be underlined that the exclusive assessment of treatment response by RECIST criteria often provides ambiguous clinical information in defining SD patients [30]. In order to obtain more reliable information, some studies have attempted to integrate cfDNA evaluation with RECIST but, at present, results have been inconclusive [25,29]. In agreement with Lee et al. [25], our findings suggest that cfDNA might help clinicians select patients at high risk of early relapse, likely allowing them to benefit from alternative therapeutic interventions. 

Concomitantly, we also investigated the prognostic role of CTC number in this cohort of patients. Unexpectedly, we observed an inverse relationship between baseline CTCs and OS. Our results contrast with the majority of reported data in NSCLC which shows that patients treated with chemotherapy had worse OS and PFS with a higher CTC number [18,31,32,33,34,35,36]. Only one study by Juan et al. [37] reported similar findings in a group of advanced NSCLC treated with docetaxel and gemcitabine. In particular, the authors found a longer median PFS and OS in patients with an increased CTC number (CTCs ≥ 2/7.5 mL) [37]. The authors concluded that a possible explanation could be due to the particular cohort of patients enrolled (high number of patient with PS = 2) and the technique used for the CTC enumeration (CellSearch system) leading to CTC underestimation. To date, the most widely used systems to enumerate CTCs are based on epithelial cell adhesion molecule (EpCAM)-based immunomagnetic techniques, such as CellSearch which is also the only FDA approved methodology. However, only about 35% of metastatic patients have EpCAM-positive CTCs [38] and evidence supports the fact that CTCs are a heterogeneous population consisting of cells with different phenotypes [39,40]. In the present study, we used a physical assay that isolates non-hematologic circulating cells by size, irrespective of cell surface markers. Moreover, filtration-based devices have a higher sensitivity to enrich the CTC subpopulations [16,31] and they also allow the detection of circulating tumor microemboli, defined as clusters of CTCs ≥ 3 [31,33,41], undetectable by CellSearch. Indeed, we found higher number of median CTCs at baseline (6/3 mL of blood) compared to previous studies using EpCAM-based techniques [31,32,33,34,36], reporting median CTC number ranging from one to six in a double volume of blood (7.5 mL). On the basis of the above considerations and present findings, we can hypothesize that a higher baseline number of heterogeneous CTC populations might exhibit different responsiveness to chemotherapy and that a higher fraction of more sensitive CTCs might have been present in the blood of patients with a better outcome (CTCs > 6/3 mL). Indeed, similar to previous studies, we observed a reduction of the CTC number following chemotherapy in PR and SD, compared to PD patients. This reduction, suggestive of treatment efficacy, might indicate that patients with better survival show a prevalence of chemo-sensitive CTC subpopulation. Likewise, the cfDNA levels progressively decrease during treatment with chemotherapy in PD, SD and PR patients, although this is not statistically significant. To date, few studies evaluated cfDNA variation during chemotherapy and reported discordant results [23,25,42,43]. Our findings were similar to those reported by Kumat et al. [42] in which cfDNA levels decreased among PD, SD and PR patients and also by Gautshi et al. [43] who observed a cfDNA increase in PD patients. Hence, we can speculate that both CTCs and plasma cfDNA may play a role as predictive biomarkers of chemotherapy efficacy. This hypothesis holds particularly true in the subsets of patients reporting SD at BOR by assuming that a higher risk of early death may be expected in patients with high cfDNA values and a low CTC number. This behavior further supports the assumption that cfDNA and CTCs have an independent prognostic role which is in line with their different mechanisms of release from the tumor into circulation; cfDNA is mainly released by a passive mechanism (tumor cell death) whereas CTCs are spread through an active process (tumor aggressiveness).

## 4. Materials and Methods

### 4.1. Patients’ Enrollment

Seventy-three patients with advanced NSCLC and eligible for first-line chemotherapy were enrolled into a prospective study at the Lung Cancer Unit, IRCCS AOU San Martino-IST, Genova, Italy [44].

The inclusion criteria were pathologically confirmed NSCLC stage IIIB-IV, no previous systemic treatment, and a performance status (ECOG PS) of 0–2. All the patients underwent a CT-scan of the chest and abdomen prior to any treatment, concomitantly with baseline blood drawing; patients with known or suspected brain metastases underwent a baseline brain scan as well. Patients were treated with first-line platinum-based chemotherapy with pemetrexed for the adenocarcinoma or gemcitabine for the squamous histology; if patients were deemed unfit for cisplatin (advanced age, comorbidities, and impaired renal function), carboplatin was employed in its place [45]. Patients receiving pemetrexed in combination with a platinum-derivate were eligible for maintenance with single-agent pemetrexed after four cycles, provided that disease control or response were achieved and the treatment was well tolerated; during maintenance, CT-scans were performed with the same timing as combination treatment. CT-scans were performed at baseline and after every two cycles of chemotherapy for response assessment, concomitantly with peripheral blood drawing for cfDNA and CTC analysis. Radiological response was assessed using RECIST *v.*1.1 to measure variations in tumor size. The present study has been approved by the Local Ethics Committee (ID#TrPo11.003; IRCCS AOU San Martino-IST) and informed written consent was obtained from each patient.

### 4.2. Circulating Free DNA Isolation and Quantification

Five mL of peripheral blood were collected in ethylenediamine tetraacetic acid (EDTA)-containing tube and plasma was isolated by two-steps of centrifugation at 1600 rpm for 15 min. cfDNA was extracted from 400 μL of the resulting plasma using the QIAamp DNA Blood Mini Kit (Qiagen, Hilden, Germany) according to the manufacturer’s protocol. The quantification of cfDNA was performed by quantitative PCR (qPCR) method, using *hTERT* single copy gene (Thermo Fisher Scientific, Waltham, MA, USA). The qPCR reaction was carried out in a final volume of 10 μL consisting in: 5 μL of TaqMan Universal Mastermix (Thermo Fisher Scientific), 1 μL of assay and 4 μL of cfDNA on RealPlex2 system (Eppendorf, Hamburg, Germany). Each plate included positive and negative controls. The calibration curve was calculated based on a dilution series of a standard DNA (Promega, Madison, WI, USA): 1, 10, 100, 1000, 10,000, 100,000 copy number (3.3 pg of DNA = 1 gene copy). Each sample was run in duplicate and the final concentration, means as copy number, was calculated by interpolation of the mean of cycle threshold (CT) values with the calibration curve.

### 4.3. Circulating Tumor Cell Isolation

**Figure 3:**
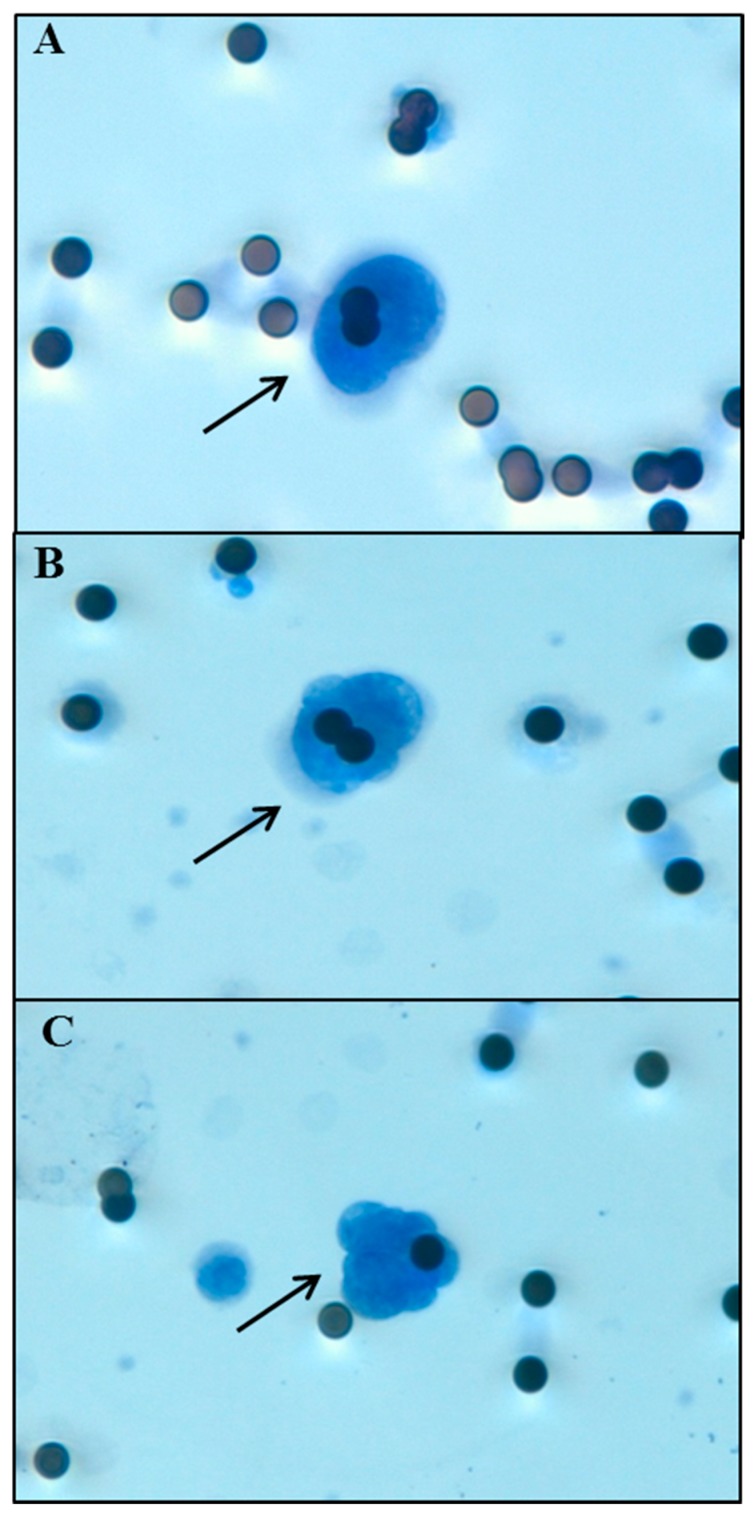
Morphological analysis of NSCLC cells entrapped on a ScreenCell Cyto device filter. The black arrows indicate non-hematologic circulating cells with malignant features. (**A**) Adenocarcinoma (H&E staining, magnification 40×); (**B**) Squamous cell carcinoma (H&E staining, magnification 40×); (**C**) Microemboli (H&E staining, magnification 40×). Micropores dimension: 7.5 ± 0.36 μm.

Circulating cells were isolated from whole peripheral blood of NSCLC patients by the filtration-based device ScreenCell Cyto (ScreenCell, Paris, France) according to manufacturer’s protocol. Briefly, 3 mL of blood are mixed in an appropriate buffer to lyse erythrocytes and fix leukocytes and non-hematologic circulating cells. Circulating cells were separated through a microporous membrane filter allowing only cells larger than the pores (7.5 ± 0.36 μm) to be retained on the membrane. The filter was then released on a slide, stained with haematoxylin-eosin (H&E) and observed under a light microscope. The isolated non-hematologic circulating cells with malignant features were defined as CTCs and morphologically identified and enumerated according to the following criteria: nuclear size greater than or equal to 20 μm, high nuclear/cytoplasmic ratio (≥0.75), dense hyperchromatic nucleus, and irregular nuclear membrane as already reported by Freidin and colleagues (Figure 3) [46].

### 4.4. Statistical Methods

Continuous variables (age at first cycle and time interval between diagnosis and first chemotherapy administration) were described using median and inter-quartile range, while categorical factors (gender, histotype, metastatic status, and ECOG PS) were reported in terms of absolute and relative frequencies.

The prognostic role of cfDNA and CTCs on PFS and OS were explored using the Kaplan–Meier methodology and differences in survival probabilities were statistically assessed through the log-rank test, after dichotomizing cfDNA and CTCs according to their median values, due to the relatively small sample size in order to guarantee comparable subgroup size and similar precision of parameter estimates. The prognostic effect of cfDNA and CTCs adjusted for potential imbalances in baseline patients’ characteristics (age at start of chemotherapy, gender, histology, metastatic status, and ECOG PS) was estimated through Cox regression modeling. The HR, and corresponding 95% CL, was used as a measure of relative risk of relapsing or dying during the follow-up period. Statistical significance of the HR was assessed using the likelihood ratio test [47].

Finally, the relationship between BOR, cfDNA and CTC levels at the end of the second cycle was estimated using multiple linear regression after log-transformation of the biomarker measurements. Regression results were adjusted for potential imbalances in baseline individual characteristics (age at first cycle, gender, histology, metastatic status, and ECOG PS), which included log-transformed cfDNA and CTCs at first cycle. For all statistical comparisons, a *p*-value ≤ 0.05 was considered statistically significant.

All data analyses were performed using Stata (StataCorp. Stata Statistical Software, release 13.1 Statistical Software. College Station, TX: StataCorp LP, 2013).

## 5. Conclusions

Lung cancer prognosis remains a top priority worldwide and efforts to identify non-invasive biomarkers to improve the current imaging tools and predict treatment efficacy need to be addressed.

To the best of our knowledge, this is the first study assessing the prognostic role of cfDNA and CTCs, in separate and joint analyses, in a cohort of advanced NSCLC treated with first-line chemotherapy regimens. cfDNA demonstrated a more reliable marker than CTCs, considering the overall population. Moreover, the results observed in the subgroup of SD subjects that identified patients at a higher risk of shorter survival, addressed the relevance of associating imaging techniques with cfDNA and CTC estimations; this helps clinicians identify patients deserving additional/alternative therapeutic interventions.

The standardization of the procedures remains challenging due to different methods of extraction and quantification of cfDNA and CTC detection, although we assume that, concerning CTCs, a filtration-based technique might represent a better isolation tool to enrich subpopulations with diverse phenotypes. Large and methodologically uniform studies are required to confirm these data and better elucidate the significance of circulating biomarkers in treated NSCLC patients.

## Figures and Tables

**Table 1:** Patients’ clinical characteristics.

**Characteristics**	
**Number of patients**	73
**Age at first cycle (years)**	
Median	67
Range	44–80
**Gender *n* (%)**	
Male	50 (68.5%)
Female	23 (31.5%)
**Histology *n* (%)**	
Adenocarcinoma	59 (80.8%)
Squamous cell carcinoma	14 (19.2%)
**Metastases *n* (%)**	
M1a	23 (31.5%)
M1b	50 (68.5%)
**Evidence of Brain Metastases *n* (%)**	
Yes	19 (26%)
No	54 (74%)
**ECOG PS *n* (%)**	
0	21 (28.8%)
1–2	52 (71.2%)
**Smoking status *n* (%)**	
Current smoker	31 (42.5%)
Former smoker	36 (49.3%)
Never smoker	6 (8.2%)

Abbreviations: ECOG PS: Eastern Cooperative Oncology Group Performance Status.

**Table 2:** Eighteen-month overall survival estimated through the Kaplan–Meier methodology.

Factors & Levels	Number of Patients	Number of Deaths (%)	18-Month OS		*p-*Value
			Prob.	95% CL	
Baseline cfDNA					
≤96.3	37	31 (83.8)	0.38	0.23–0.53	0.019
>96.3	35	33 (94.3)	0.09	0.02–0.21	
Not evaluable	1	1 (100.0)	-	-	
Baseline CTCs					
≤6	39	34 (87.2)	0.18	0.08–0.31	0.402
>6	34	31 (91.2)	0.29	0.15–0.45	
Age at first cycle					
≤67 years	37	31 (83.8)	0.32	0.18–0.48	0.050
>67 years	36	34 (94.4)	0.14	0.05–0.27	
Gender					
Male	50	44 (55.0)	0.24	0.13–0.36	0.589
Female	23	22 (95.7)	0.22	0.08–0.40	
Histology					
Adenocarcinoma	59	52 (88.1)	0.24	0.14–0.35	0.546
Squamous cell carcinoma	14	13 (92.9)	0.21	0.05–0.45	
Metastases					
M1a	23	22 (95.6%)	0.22	0.08–0.40	0.763
M1b	50	43 (86.0%)	0.24	0.13–0.36	
Evidence of Brain Metastases					
Yes	19	18 (94.7)	0.16	0.04–0.35	0.243
No	54	47 (87.0)	0.26	0.15–0.38	
ECOG PS					
0	21	16 (76.2)	0.29	0.12–0.48	0.202
1–2	52	49 (94.2)	0.21	0.11–0.33	
*Whole sample*	*73*	*65 (89.0)*	*0.23*	*0.14–0.34*	

Metastases M1a: thoracic district (the single stage IIIB patient was included among the group with thoracic disease); Metastases M1b: extra-pulmonary district; Prob.: 18-month survival probability estimate; 95% CL: 95% confidence limits for Prob; *p*-value: *p*-value of the log-rank test. Abbreviations: CL: confidence limits; ECOG PS: Eastern Cooperative Oncology Group; OS: Overall survival.

**Table 3:** Separate and joint effects of Cell-free DNA (cfDNA) and circulating tumor cells (CTCs) on progression free and overall survival probabilities estimated using the Cox multiple regression modeling.

**Factors & Levels**	**Separate Effect**					
	**PFS**			**OS**		
	**HR**	**95% CL**	***p*-Value**	**HR**	**95% CL**	***p*-Value**
Baseline cfDNA			0.040			0.006
≤96.3	1.00			1.00		
>96.3	1.70	1.02–2.83		2.14	1.24–3.68	
Baseline CTCs			0.256			0.158
≤6	1.00			1.00		
>6	0.75	0.46–1.23		0.68	0.40–1.16	
**Factors & Levels**	**Joint Effect**					
	**PFS**			**OS**		
	**HR**	**95% CL**	***p*-Value**	**HR**	**95% CL**	***p*-Value**
Baseline cfDNA			0.075			0.012
≤96.3	1.00			1.00		
>96.3	1.63	0.95–2.78		2.03	1.17-3.52	
Baseline CTCs			0.629			0.385
≤6	1.00			1.00		
>6	0.88	0.52–1.48		0.79	0.45–1.36	

Abbreviations: HR: hazard ratio adjusted for age at first cycle, gender, histology, metastatic status (thoracic/extra-pulmonary districts and evidence of brain metastases), and ECOG PS; 95% CL: 95% confidence limits for HR; OS: overall survival; PFS: progression-free survival; *p*-value: *p*-value of the likelihood ratio test.

**Table 4:** Separate and joint effects of cfDNA and CTCs on overall survival probabilities estimated using the Cox multiple regression modeling in patients with stable disease at best overall response.

Factors & Levels	Separate Effect			Joint Effect		
	OS			OS		
	HR	95% CL	*p*-Value	HR	95% CL	*p*-Value
Baseline cfDNA			0.047			0.153
≤96.3	1.00			1.00		
> 96.3	2.32	1.01–2.53		1.87	0.79–4.46	
Baseline CTC			0.058			0.194
≤6	1.00			1.00		
>6	0.47	0.22–1.03		0.59	0.26–1.32	

Abbreviations: HR: hazard ratio adjusted for age at first cycle, gender, histology, metastatic status (thoracic/extra-pulmonary districts and evidence of brain metastases), and ECOG PS; 95% CL: 95% confidence limits for HR; OS: overall survival; PFS: progression-free survival; *p*-value: *p*-value of the likelihood ratio test.

## Abbreviations

*ALK*	Anaplastic lymphoma kinase
BOR	Best Overall Response
cfDNA	cell-free tumor DNA
CL	Confidence Limits
CT	Cycle Threshold
CTCs	Circulating Tumor Cells
CT-scan	Computed Tomography
ECOG PS	Eastern Cooperative Oncology Group Performance Status
EDTA	Ethylenediamine tetraacetic acid
*EGFR*	Epidermal growth factor receptor
EpCAM	Epithelial Cell Adhesion Molecule
GM	Geometrical Mean
H&E	Haematoxylin-Eosin
HR	Hazard Ratio
*hTERT*	human Telomerase Reverse Transcriptase
NSCLC	Non-small cell lung cancer
OS	Overall Survival
PD	Progressive Disease
PFS	Progression-Free Survival
PR	Partial Response
qPCR	quantitative PCR
RECIST	Response Evaluation Criteria in Solid Tumors
SD	Stable Disease
